# A Review on the Coalescence of Confined Drops with a Focus on Scaling Laws for the Growth of the Liquid Bridge

**DOI:** 10.3390/mi14112046

**Published:** 2023-10-31

**Authors:** Sangjin Ryu, Haipeng Zhang, Udochukwu John Anuta

**Affiliations:** 1 Department of Mechanical and Materials Engineering, University of Nebraska-Lincoln, Lincoln, NE 68588, USA; hzhang4@ccny.cuny.edu (H.Z.);; 2 Nebraska Center for Materials and Nanoscience, University of Nebraska-Lincoln, Lincoln, NE 68588, USA

**Keywords:** Ohnesorge number, contact angle, surface wettability, multiphase flow, Hele-Shaw cell, surface tension, viscous regime, inertial regime

## Abstract

The surface–tension-driven coalescence of drops has been extensively studied because of the omnipresence of the phenomenon and its significance in various natural and engineering systems. When two drops come into contact, a liquid bridge is formed between them and then grows in its lateral dimensions. As a result, the two drops merge to become a bigger drop. The growth dynamics of the bridge are governed by a balance between the driving force and the viscous and inertial resistances of involved liquids, and it is usually represented by power–law scaling relations on the temporal evolution of the bridge dimension. Such scaling laws have been well-characterized for the coalescence of unconfined or freely suspended drops. However, drops are often confined by solid or liquid surfaces and thus are a different shape from spheres, which affects their coalescence dynamics. As such, the coalescence of confined drops poses more complicated interfacial fluid dynamics challenges compared to that of unconfined drops. Although there have been several studies on the coalescence of confined drops, they have not been systematically reviewed in terms of the properties and geometry of the confining surface. Thus, we aim to review the current literature on the coalescence of confined drops in three categories: drop coalescence on a solid surface, drop coalescence on a deformable surface, and drop coalescence between two parallel surfaces with a small gap (i.e., Hele-Shaw cell), with a focus on power–law scaling relations, and to suggest challenges and outlooks for future research on the phenomena.

## 1. Introduction

The surface–tension-driven merge, or coalescence, of drops is a fascinating interfacial fluid dynamics phenomenon. When two liquid drops come into contact with each other, a liquid bridge or neck is formed between these drops, and then it grows rapidly driven by the surface tension between the drops and their outer fluid. In the end, the two drops completely merge, forming a larger drop. This process is seen not only in daily life, but also in various industrial processes. Examples include oil drops in salad dressing, water droplets in a cloud, rain drops hitting a water puddle, paint droplets in spray painting, and so on. In these common examples, drops are free of confinement. As such, the coalescence of two unconfined drops has been well studied for understanding the basic principles of drop coalescence [1,2,3,4].

**Figure 1:**
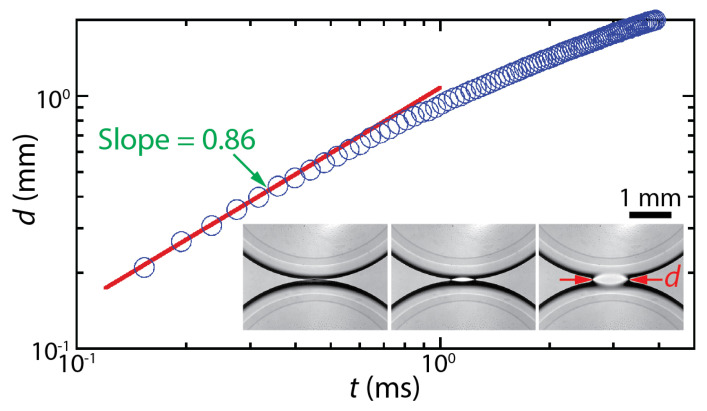
The temporal growth of the liquid bridge width (*d*) between two corn syrup drops coalescing in mineral oil in a Hele-Shaw cell of a depth of *D* = 1 mm [6,7,8]. This case belongs to the two-phase drop coalescence with two confining surfaces category shown in Figure 2. The viscosities of the drop and the outer liquid were *μ_d_* = 20.6 mPa·s and *μ_o_* = 19.1 mPa·s, respectively. Since the slope of the fitted red line was 0.86, the bridge growth followed a scaling law of *d*~*t*^0.86^ in the early times of the coalescence. (Inset) Sequential top-view images of the growing liquid bridge. Time interval: 0.408 ms.

The dynamics of drop coalescence are usually represented by the growth dynamics of the liquid bridge by how the diameter or width of the bridge (*d*) increases in time (*t*) [4,5]. Drop coalescence is captured using high-speed videography due to the extremely fast occurrence of the event, and then *d* is measured from the captured images [1]. For example, the inset of Figure 1 shows two corn syrup drops coalescing in mineral oil confined in a Hele-Shaw cell with a depth of *D* = 1 mm [6,7,8]. The coalescence process was imaged at a frame rate of 49,000 fps (Δ*t* ≈ 0.02 ms). Upon the initial contact of the drops, a liquid bridge was formed, and then the bridge grew extremely fast. When measured *d* values are plotted with *t* in the log–log scale (Figure 1), a linear trend of *d* in the early times suggests a power–law scaling relation of *d*~*t^n^*. Here, *n* is the scaling exponent, which corresponds to the slope of the linear trend in the log–log plot. In the shown example, *n* was determined to be 0.86 based on curve fitting (the red line in Figure 1). After the early times, the growth of the liquid bridge became slower (i.e., d*d*/d*t* decreased with *t*), and thus the slope decreased for the later times. Since *n* values are related to the growth dynamics of the liquid bridge during drop coalescence, studies on drop coalescence aim to determine and understand the power–law dependence of the bridge growth with respect to time (i.e., *d*~*t^n^*).

For the coalescence of unconfined or freely suspended drops, the phenomenon is governed by balance between capillary, viscous, and inertial forces. Depending on whether inertial or viscous force is the dominant resistance to capillary force, different regimes with different *n* values can be identified. For the viscous regime, the linear growth of the liquid bridge with time was observed (i.e., *d*~*t*^1^). This viscous regime was followed by the inertial regime for which the following power–law scaling relation was found: *d*~*t*^1/2^ [9,10,11,12,13,14,15,16]. In addition to these two regimes, a third regime named the inertially-limited-viscous regime was recently found, where the coalescence is governed by a balance between all three forces [17].

Drop coalescence frequently occurs while drops are bound or confined by a surface or surfaces. For instance, drops in a microfluidic device interact with each other while confined by the two parallel walls of the microchannel. During ink-jet printing, ink droplets merge on paper. Similarly, water drops from condensation coalesce on a cooling solid surface. Enhanced oil recovery involves coalescence of oil drops in underground pores. The surface confinement makes the process of drop coalescence more complicated. For instance, when drops coalesce on a substrate with a small contact angle (*θ*), their coalescence dynamics is known to be significantly affected by viscous resistance imposed by the substrate, and as *θ* increases, the coalescence dynamics approaches that of freely suspended drops [18]. In other words, the coalescence dynamics of drops depends on the surface wettability of the substrate. In addition, the existence and motion of a three-phase contact line can complicate the coalescence phenomenon [19].

**Figure 2:**
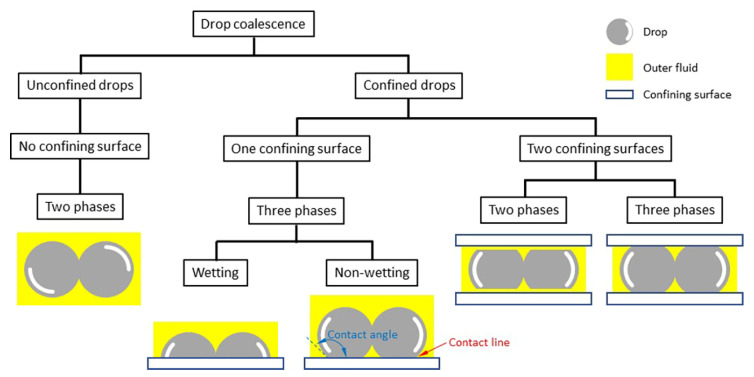
Categories of drop coalescence.

Figure 2 shows how we categorized types of drop coalescence. The phenomena can be divided largely into two groups depending on whether any confining solid surfaces are involved. When drops merge free of any confining surfaces, this can be categorized as unconfined drop coalescence. In this case, drops coalesce in an outer fluid, which can be either a gas or an outer immiscible liquid. Thus, in this category, only two phases are involved. Since the cross-section of the liquid bridge is circular in this category, the growth of the bridge is characterized by measuring the diameter (*d*) or radius (*r*) of the bridge. As aforementioned, most previous studies investigated the coalescence of unconfined or freely suspended drops [1,3,4,20].

In contrast to the unconfined drop coalescence category, drops can be confined by solid surfaces, which is categorized as the confined drop coalescence [21,22]. It is possible for multiple solid surfaces to be involved in drop coalescence, but practically we will consider two subcategories. The first subcategory is the coalescence of drops confined by a common solid surface on which the drops rest. In this case, three different phases exist: a drop liquid, an outer fluid that can be either a gas or an immiscible liquid, and a solid surface. As such, a contact line exists between the drops and the surface, and its pinning and depinning can affect drop coalescence.

Additionally, the shape of drops depends on the *θ* of the drop on the surface in the first subcategory. When *θ* < 90°, which means that the drop liquid can wet the solid surface, the initial contact between the drops occurs at a point on the contact line. Thus, the created liquid bridge is also in contact with the surface. Consequently, the cross-sectional shape of the bridge is not axisymmetric for this case, and characterizing the bridge growth requires measuring two characteristic dimensions of the bridge: the width (*d*) or half-width (*r*) of the bridge measured in the top view, and the height (*h*) of the bridge measured in the side view. When *θ* > 90°, the drop liquid cannot wet the solid surface, and thus the initial contact likely occurs at a point on the equator of the drops. As a result, the liquid bridge is created away from the surface and then grows toward the surface. The dynamics of the bridge growth is expected to be similar to that of the unconfined drops until the bridge touches the surface. It is also expected that the outer fluid can be trapped between the liquid bridge and the surface. Since the interaction of the liquid bridge with the surface differs depending on *θ*, the subcategory of the one confining surface cases can be further divided into the wetting and not-wetting cases, as shown in Figure 2.

In the second subcategory of the confined drop coalescence, drops are confined between two parallel solid surfaces with a small gap. Such geometries are called Hele-Shaw cells, and the depth of the Hele-Shaw cell (*D*) is smaller than, or comparable to, the size of the drops. For instance, drops formed in a microchannel fall into this category. In contrast to the first subcategory, the Hele-Shaw cell provides an advantage that drops and their liquid bridge are symmetrical with respect to the midplane of the cell. When a Hele-Shaw cell is filled with a liquid and drops of an immiscible liquid are formed and coalesce in the outer liquid, drops do not make direct contact with the confining surface due to the existence of a thin layer of the outer liquid between the drop and the surface [8,23]. Thus, drop coalescence involves only two phases despite the solids surface (i.e., two-phase coalescence), and the surface wettability of the surface does not matter. In contrast, when the Hele-Shaw cell is filled with a gas and drops are created and merge in the cell, a contact line exists, and thus the drop coalescence involves three phases (i.e., three-phase coalescence). In this case, the surface wettability of the solid surface determines the shape of the drop.

In this review paper, we will mainly focus on experimental studies on the coalescence of confined drops and found power–law scaling relations for the bridge growth. Among drop coalescence cases with confining surfaces, cases with an axisymmetric confining surface (e.g., drop coalescence in a round tube) and three-phase coalescence in a Hele-Shaw cell are not considered. This review paper is structured as follows: drop coalescence with one rigid confining surface in Section 2, drop coalescence with one deformable confining surface in Section 3, drop coalescence between two parallel confining surfaces in Section 4, relevant computational studies in Section 5, and summary and outlook in Section 6.

## 2. Coalescence of Drops on a Rigid Surface

The shape of drops resting on a solid surface depends on the surface wettability of the surface, which is represented by *θ*. When *θ* < 90°, drops have a spherical cap shape with a polar angle smaller than 90°, and the initial contact between them occurs where the contact lines of the two drops meet. Thus, the liquid bridge grows while in contact with the surface, and its growth dynamics appear to be affected by the contact angle between the bridge and the surface and the pinning and depinning of the contact line. In contrast, when *θ* > 90°, drops are in the spherical cap shape with the polar angle greater than 90°, and the initial contact between them occurs where the equators of the two drops meet. Thus, the liquid bridge is formed away from the surface, and its growth dynamics are not affected by the surface until it touches the surface. Therefore, understanding the coalescence dynamics of two sessile drops resting on a solid surface requires consideration of the surface wettability of the surface. This section summarizes related previous studies in the order of increasing *θ*.

### 2.1. Wetting Cases (θ < 90°)

**Figure 3:**
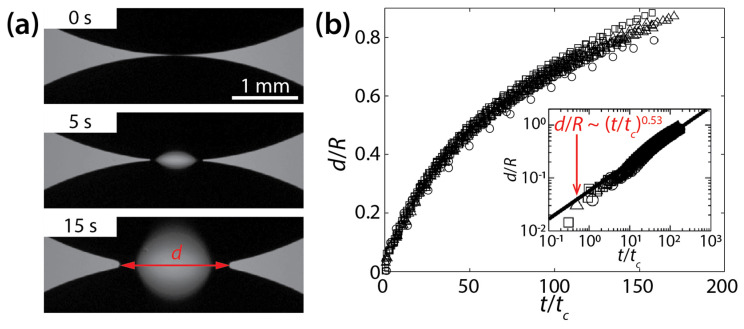
(**a**) Coalescence of silicone oil drops (*ν_d_* = 10^−3^ m^2^/s, *ρ_d_* = 970 kg/m^3^, and *σ* ≈ 20 mN/m) on a wettable polystyrene substrate (*θ* ≈ 0°) (top view). (**b**) A plot of *d*(*t*) for different kinematic viscosities (○: 10^−4^ m^2^/s, ☐: 3.5 × 10^−4^ m^2^/s, △: 10^−3^ m^2^/s). Inset: log–log scale plot. Here, *t_c_* = *μ_d_R*^4^/*σH*^3^. Adapted with permission from [24]. Copyright 2006 by the American Physical Society.

Ristenpart et al. studied the early-time coalescence dynamics of silicone oil drops on a polystyrene surface in air [24]. The tested silicone oils had similar surface tension coefficient values of *σ* ≈ 20 mN/m in the air, despite different kinematic viscosities (*ν_d_* = 10^−4^, 3.5 × 10^−4^, and 10^−3^ m^2^/s). Here, the subscript *d* stands for drop. The density of the oils was *ρ_d_* = 970 kg/m^3^ [18]. When two drops in approximate volume of 20 μL were placed on the surface, they spread radially because *θ* ≈ 0°, and eventually the initial heights of the drop (*H*) became much smaller than their radii *R* (i.e., *H* << *R*). Here, *R* is the radius of the drop measured in the top-view image. Upon the contact of the drops, the liquid bridge was formed, and it grew laterally while the drops were coalescing (Figure 3a).

The coalescence process was found to be viscously dominated because, in general, less viscous drops coalesced more rapidly. The process was dependent on the geometry of the drops because the found characteristic time scale included *H* and *R* (*t_c_* = *μ_d_R*^4^/*σH*^3^). Although there was a significant variance in *d*(*t*), even with the same liquid, the results collapsed well when the dimensionless width of the bridge (*d*/*R*) was plotted against *t*/*t_c_* (Figure 3b). When plotted in the log–log scale, the results followed a scaling law of *d*/*R*~(*t*/*t_c_*)^0.53^ (the inset of Figure 3b). The found power–law exponent was confirmed based on the mass conservation on the growing liquid bridge as the analysis yielded *d*~(*t*/*t_c_*)^1/2^. The scaling law of Ristenpart et al. [*d*~(*σH*^3^*t*/*μ_d_R*^2^)^1/2^] sets a lower bound (i.e., a limiting case of *θ*→0°) for the coalescence dynamics of drops on a solid surface.

**Figure 4:**
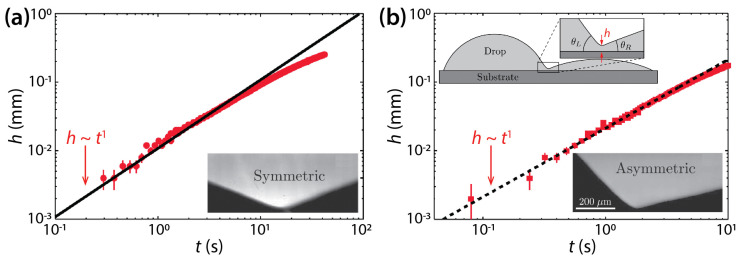
(**a**) Coalescence of symmetric silicon oil drops (*μ_d_* = 12.2 Pa·s, *ρ_d_* = 975 kg/m^3^, and *σ* = 21 mN/m) on glass (*θ* = 22°). Inset: A magnified side-view image of the liquid bridge. (**b**) Coalescence of asymmetric drops (*θ_L_* = 46° and *θ_R_* = 13°). Top inset: Schematics of drops (side view). Bottom inset: A magnified side-view image of the liquid bridge for the asymmetric case. Adapted with permission from [25]. Copyright 2012 by the American Physical Society.

Hernández-Sánchez et al. investigated coalescence of silicone oil drops (*μ_d_* = 0.974 Pa·s or 12.2 Pa·s, *ρ_d_* = 975 kg/m^3^, and *σ* = 21 mN/m) on glass [25]. Although they studied symmetric drops as shown in Figure 4a, as Ristenpart et al. did, there are two differences. First, the *θ* of Hernández-Sánchez et al. (*θ* = 22°) was greater than that of Ristenpart et al. Second, they studied the dynamics of *h*, not *d*. Hernández-Sánchez et al. found that the side-view profile of the bridge grew self-similarly with a scaling law of *h*~*t* (Figure 4a). They also confirmed the hypothesis of Ristenpart et al.—that the coalescence of sessile drops on a single solid surface is “governed by liquid flux from the drop into the bridge”. Also, they revealed that the rate of the vertical growth of the liquid bridge scaled with *θ*^4^ (i.e., d*h*/d*t*~*θ*^4^).

Also, Hernández-Sánchez et al. investigated coalescence of asymmetric drops in terms of *θ*. When *θ_L_* ≠ *θ_R_*, where *θ_L_* (= 46°) and *θ_R_* (= 13°) are the contact angles of the left- and right-hand side drops, the minimum height point of the liquid bridge moved towards the drop with lower *θ*. As shown in Figure 4b, despite the asymmetry in *θ*, *h* increased linearly with *t*, similarly to the symmetric case.

**Figure 5:**
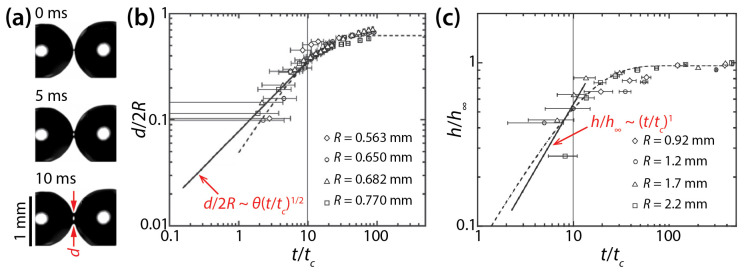
(**a**) Coalescence of diethylene glycol (DEG) drops (*μ_d_* = 0.161 Pa·s, *ρ_d_* = 1130 kg/m^3^, and *σ* = 47 mN/m) on a wettable silicon surface (*θ_a_* = 35°, and *θ_r_* = 25°) (top view). (**b**) Log–log scale plot of *d*(*t*) for different drop sizes. (**c**) Log–log plot of *h*(*t*) for different drop sizes. Here, *t*_c_ = *μ_d_R*/*σ*. Adapted with permission from [26].

Narhe et al. derived the following scaling laws based on balance between the Laplace pressure and viscous stress in the bridge: *h*/*h*_∞_~*t*/*t_c_* and *d*/2*R*~*θ*(*t*/*t_c_*)^1/2^, where *h*_∞_ is the *h* value at the end of the coalescing process and *t_c_* = *μ_d_R*/*σ*. They tested their scaling laws by examining the coalescence of diethylene glycol (DEG) drops (*μ_d_* = 0.161 Pa·s, *ρ_d_* = 1130 kg/m^3^, and *σ* = 47 mN/m) on silicon surface (Figure 5a) [26]. The *θ* of the drop was determined to be 30° by averaging the advancing angle (*θ_a_* = 35°) and the receding angle (*θ_r_* = 25°). As Figure 5b,c show, the early time growth of *d* and *h* somewhat followed the found scaling laws, but the quality of agreement is slightly questionable because of the lack of data points near the initiation of the coalescence (*t*/*t_c_* ≤ 1) and large error bars. The later times of the coalescence could be well-represented by exponential growth. Later, Lee et al. compared studies of Ristenpart et al. [24] and Narhe et al. [26] and found that the scaling law of Narhe et al. did not agree with that of Ristenpart et al. because the former became *d*~(*σH*^2^*t*/*μ_d_R*)^1/2^ as *θ* approached 0° [18].

Lee et al. examined coalescence of sessile DEG drops (*μ_d_* = 38.5 mPa·s, *ρ_d_* = 1118 kg/m^3^, and *σ* = 43.16 mN/m) on indium tin oxide (ITO)-coated solid substrates of different wettabilities (*θ* = 10°, 24°, 27°, and 56°) [18]. The volume of drops ranged 0.524–7.24 μL, and the initial radius seen from the top was *R* = 110.5–240 μm. The Reynolds number, defined as *ρ_d_Rσ*/*μ_d_*^2^, ranged from 3.6 to 7.8, which was comparable to that of Ristenpart et al. [24].

Using the lubrication approximation, Lee et al. found the characteristic time scale for the early-time growth of the bridge to be *t_c_* = 3*μ_d_R*/4*σ*tan^4^*θ* and the scaling law of the half-width of the bridge to be *r*~(*σh_o_*^3^/*μ_d_*)^1/4^*t*^1/4^, where *h_o_* is the initial bridge height. The found time scale was better than *μ_d_R*/*σ* because data sets of *h*(*t*) approached closer to each other when *t* was normalized with *t_c_* = 3*μ_d_R*/4*σ*tan^4^*θ* than with *t_c_* = *μ_d_R*/*σ*. However, it should be noted that the data sets still showed significant disagreement despite normalization with *t_c_* = 3*μ_d_R*/4*σ*tan^4^*θ*. For their scaling law derivation, Lee et al. assumed that flow developing in the bridge did not affect the domain near the contact line, and thus *θ* was preserved.

For the bridge height, Lee et al. found that the scaling exponent of *h*/*R*~(*t*/*t_c_*)*^n^* was 0.51, 0.64, 0.67, and 0.86 for *θ* = 10°, 24°, 27°, and 56°, respectively. The sizes of these drops were *R* = 240 μm, 225 μm, 199 μm, and 110.5 μm, respectively. This result shows that the *n* value of *h*(*t*) increased with *θ*, which shows that the coalescence dynamics were affected by the wettability of the substrate.

**Figure 6:**
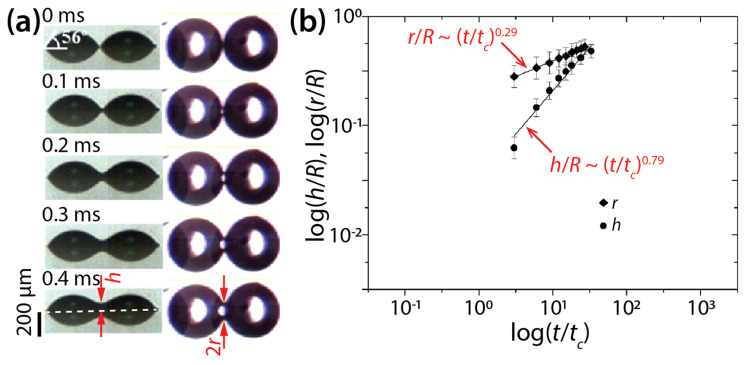
(**a**) Side- and bottom-view images of two coalescing diethylene glycol (DEG) drops (*μ_d_* = 38.5 mPa·s, *ρ_d_* = 1118 kg/m^3^, *σ* = 43.16 mN/m, and *R* = 240 μm) on indium tin oxide (ITO) substrate (*θ* = 56°). (**b**) Log–log scale plot of *r*(*t*) and *h*(*t*). Here, *t_c_* = 3*μ_d_R*/4*σ*tan^4^*θ*. Adapted with permission from [18]. Copyright 2012 American Chemical Society.

Lee et al. also conducted simultaneous imaging of the side- and top-views of DEG drops with *θ* = 56° and *R* = 240 μm (Figure 6) and found scaling laws of *h*/*R*~(*t*/*t_c_*)^0.79^ and *r*/*R*~(*t*/*t_c_*)^0.29^. Regarding *r*(*t*), their measured *n* value (0.29) was close to that of their derived scaling law (0.25). Regarding *h*(*t*), the found *n* values differed slightly (0.86 and 0.79) for the same *θ* of 56°, which suggests variance in *n* for an identical condition.

**Figure 7:**
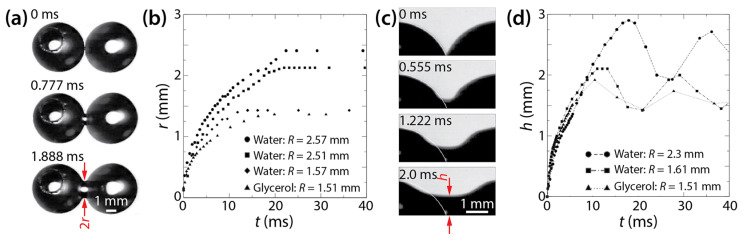
(**a**) Coalescence of two water drops on a glass surface in air (top view). (**b**) Linear scale plot of *r*(*t*). (**c**) The growth of the liquid bridge between two water drops (side view). (**d**) Linear scale plot of *h*(*t*). For distilled water, *μ_d_* = 1.07 mPa·s, *σ* = 74.0 mN/m, *ρ_d_* = 1000 kg/m^3^, *θ_a_* = 64°, and *θ_r_* = 58°. For glycerol solution, *μ_d_* = 5.7 mPa·s, *σ* = 70.5 mN/m, *ρ_d_* = 1045 kg/m^3^, *θ_a_* = 56°, and *θ_r_* = 49°. Adapted with permission from [27]. Copyright 2007 by the American Physical Society.

Kapur and Gaskell investigated the coalescence of water or glycerol solution drops (*R* < 3 mm) on a glass substrate in air (Figure 7a) [27]. The properties of the used liquids are as follows: *μ_d_* = 1.07 mPa·s, *ρ_d_* = 1000 kg/m^3^, *σ* = 74.0 mN/m, *θ_a_* = 64°, and *θ_r_* = 58° for water, and *μ_d_* = 5.7 mPa·s, *ρ_d_* = 1045 kg/m^3^, *σ* = 70.5 mN/m, *θ_a_* = 56°, and *θ_r_* = 49° for glycerol solution. Despite the significant difference in *μ_d_*, their *ρ_d_*, *σ*, and *θ* were similar. The Ohnesorge number ($\mathrm{Oh}=\mu_{d}/\sqrt{2\rho_{d}\sigma R}$) was in the order of 10^−3^ and 10^−2^ for water and glycerol drops, which means that inertial effects were dominant.

Figure 7b shows that the growth of the bridge width depended on *R* and *μ_d_*, which agrees with Ristenpart et al. [24]. Using *t_c_* = *μ_d_R*/*σ*, Kapur and Gaskell found that the scaling exponent of *r*/2*R*~(*t*/*t_c_*)*^n^* was 0.42–0.57 for the early times (0 < *t* < 0.01 s). The obtained *n* values were similar to that of the drop coalescence on a completely wettable surface (*θ* ≈ 0°) of Ristenpart et al. (*n* = 0.53) [24].

As shown in Figure 7c, Kapur and Gaskell could observe drops from the side view because of large *θ*. They measured *h*(*t*) as shown in Figure 7d. It seems that the vertical growth of the liquid bridge did not depend on the size and properties of the drops because *h* showed negligible dependence on *R* and *μ_d_*, which differed from *r*. Another difference between the horizontal and vertical growth of the liquid bridge is that *h* oscillated in the later times of drop coalescence (*t* > 10 s), whereas *r* did not. This oscillation in *h* was due to the capillary wave created at the verge of coalescence. In contrast, *r* did not oscillate, presumably due to the pinning effect of the contact line.

Regarding the vertical growth of liquid bridge, Kapur and Gaskell employed the following scaling law of unconfined drop coalescence, equating *h* to the bridge radius of freely suspended drops: *h*/*R* = *c*(*t*/*t_c_*)^1/2^, where $t_{c}=\sqrt{\rho_{d}R^{3}/\sigma }$. The *c* value was found to range from 0.98 to 1.29, which agreed with those found for unconfined drops [10,11,12].

**Figure 8:**
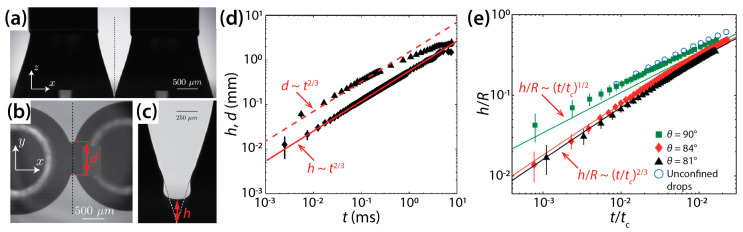
(**a**) Coalescence of two conical water drops (*σ* = 72 mN/m, *θ* = 73°) (side view). (**b**) Bottom view image of the drops. (**c**) Magnified side-view of the liquid bridge 278 μs after contact. (**d**) Log–log scale plot of *h*(*t*) and *d*(*t*) for *θ* = 73°. (**e**) Log–log scale plot of *h*(*t*) for *θ* = 81°, 84°, and 90°. Here, $t_{c}=\sqrt{\rho_{d}R^{3}/\sigma }$, where *R* was the drop radius seen from the side view. Blue circles from [29]. Adapted with permission from [28]. Copyright 2013 by the American Physical Society.

Eddi et al. characterized the effects of surface wettability on the inertial coalescence of water drops (*σ* = 72 mN/m) on a glass substrate [28]. The glass substrates were coated differently to achieve various *θ* of 73°, 81°, 84°, and 90°. For *θ* = 73°, conical drops were created as shown in Figure 8a, and thus the drops appeared as wedges in the side view. Upon contact, a thin liquid bridge was formed between the drops and then grew rapidly. This growth was accompanied by a capillary wave (Figure 8c) which suggests negligible effects of liquid viscosity. *h* was found to grow following *h*~[*σ*/*ρ_d_*(π/2 − *θ*)]^1/3^*t*^2/3^, as shown in Figure 8d. Similarly, *d* also grew with time as *t*^2/3^, as shown in Figure 8d. Despite an increase in *θ* in the hydrophilic range (*θ* < 90°), the same *n* value of 2/3 was observed for *h* (Figure 8e).

In contrast to the above hydrophilic cases, *n* was found to be 1/2 when *θ* = 90° (the green squares in Figure 8e). This scaling behavior (*h*~*t*^1/2^) is the same as that of inertial coalescence of unconfined drops (the blue circles in Figure 8e) [29]. With this *θ*, drops were hemispherical. If the glass surface mirrors the drop shape, the drop geometry becomes identical to that of two unconfined spherical drops. Although it is obvious that the glass surface imposes the no-slip boundary condition to the growing liquid bridge, such viscous effects appeared negligible because the coalescence was inertia-dominated. Based on these results, Eddi et al. suggested the following dynamics of liquid bridge growth for inertial drop coalescence: 0.89^3^*σt*^2^/*ρ_d_* = *h*^2^*R*{sin*θ* − [1 − (*h*/*R* + cos*θ*)^2^]^1/2^} for *θ* ≤ 90°. Here, *R* is the drop radius seen from the side view.

### 2.2. Non-Wetting Cases (θ > 90°)

**Figure 9:**
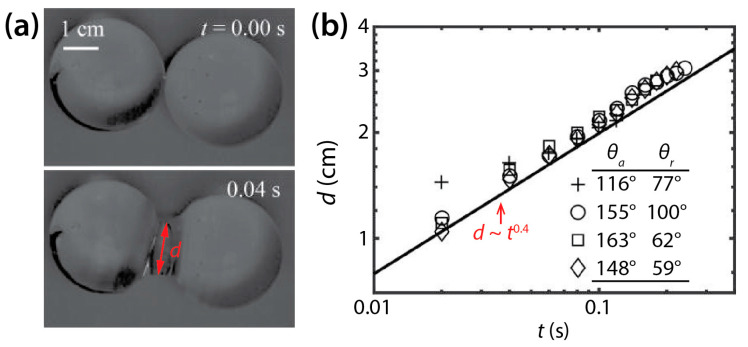
(**a**) Coalescence of two water drops (*ρ_d_* = 998 kg/m^3^, *μ_d_* = 0.998 mPa·s, and *σ* = 72 mN/m) on a non-wettable Teflon surface in air (*θ* ≈ 143°) (top view). (**b**) Log–log scale plot of *d*(*t*). Adapted with permission from [30].

In contrast to the coalescence of wetting drops on a single solid surface, the coalescence of non-wetting drops has been less studied. Recently, McCraney et al. examined the coalescence of water drops on Teflon substrates (*θ* = 115°–143°) in microgravity conditions (Figure 9a) [30]. Although coalescing water drops were different in size and shape, *d* was found to increase following *d*~*t*^0.4^ regardless of *θ*, as shown in Figure 9b.

**Figure 10:**
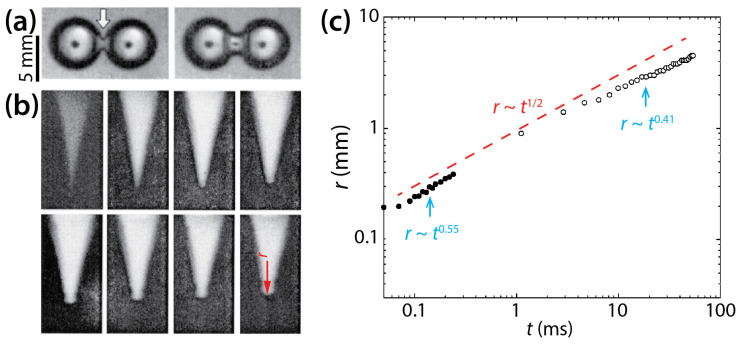
(**a**) Coalescence of two 1 g mercury drops (*ρ_d_* = 13,600 kg/m^3^, *σ* = 435 mN/m, and *R* ≈ 2.6 mm) on a non-wettable glass surface in air (*θ* ≈ 160°) (top view). Time interval: 3.5 ms. (**b**) Evolution of the liquid bridge, which is indicated by the white arrow in (**a**). Time interval: 20 μs. (**c**) Log–log scale plot of *r*(*t*). Solid and hollow markers show measurements from the early times and later times of the coalescence, respectively. The red dashed line represents a reference slope of 0.5. Adapted with permission from [9]. Copyright 2001 by the American Physical Society.

Menchaca-Rocha et al. investigated the coalescence of mercury drops (*ρ_d_* = 13,600 kg/m^3^, *σ* = 435 mN/m, and *R* = 1.2–3.0 mm) on a glass surface that was treated for minimal wetting (*θ* ≈ 160°) (Figure 10) [9]. Since the solid surface was not wettable as shown by large *θ*, the spreading of the drops was limited, and the drops are thought to have been nearly spherical. As shown in Figure 10a,b, a narrow cylindrical bridge was formed between the drops, and as it grew, capillary axial waves propagated to the undisturbed ends of the drops. The radius of the liquid bridge increased following *r*~*t*^0.55^ for the early time of coalescence and then *r*~*t*^0.41^ later (Figure 10c). The *n* value of McCraney et al. (=0.4) [30] agrees well with the *n* value for the early times of Menchaca-Rocha et al. Overall, the growth of the liquid bridge followed a scaling law of *r*~*t*^0.5^ as indicated by the red dashed line in Figure 10c. It is noticeable that the scaling exponent of 1/2 is known for the inertial coalescence regime of freely suspended drops [9,10,11,12,13,14,15,16,31]. Although the mercury drops were resting on the glass surface, the effect of the surface appeared negligible because the drops were in a spherical shape such as unconfined drops [24].

**Figure 11:**
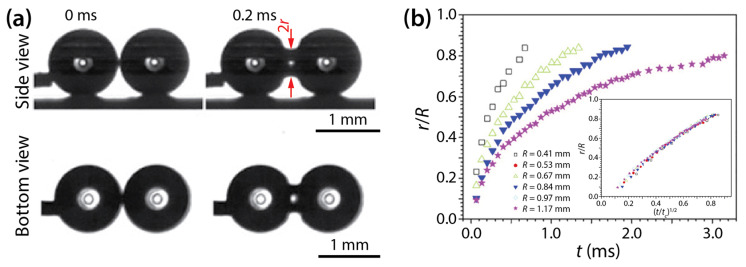
(**a**) Side- and bottom-view images of two coalescing water drops on superhydrophobic substrate (*θ* = 162–165°). (**b**) Linear scale pot of *r*(*t*) for drops of different sizes. Inset: Superposition of the data sets when normalized with *t_c_*$=\sqrt{\rho_{d}R^{3}/\sigma }$. Adapted with permission from [19].

Wang et al. examined the coalescence of water drops of ~4 μL in volume on superhydrophobic surfaces: transparent SiO_2_ surface (*θ* = 165.2 ± 2.6°) and nanostructured copper oxide surface (*θ* = 162.4 ± 2.2°) [19]. Their drops were spherical (Figure 11a), and the growth dynamics of the bridge radius was well represented by the scaling law of *r*/*R*~(*t*/*t_c_*)^1/2^, where $t_{c}=\sqrt{\rho_{d}R^{3}/\sigma }$. Since the observed scaling law is that of unconfined drops, as shown by Menchaca-Rocha et al. [9], they concluded that the liquid bridge between the drops grew similarly to that of unconfined drops well before the bridge hit the solid surface.

In their study, Wang et al. concluded that in their drop coalescence, inertial effects were dominant based on the Ohnesorge number of the drops ($\mathrm{Oh}=\mu_{d}/\sqrt{\rho_{d}\sigma R}$). According to Paulsen et al. [29], the crossover from the viscous regime or inertially-limited-viscous regime to the inertial regime happens when *r*/*R* is in the same order magnitude as Oh (i.e., *r*/*R* = *O*(Oh)). In the study of Wang et al., *R* ranged from 0.41 mm to 1.17 mm, and the range of Oh was 0.003–0.006. Since Oh << 0.1, the said transition occurred at very small *r*, which suggests that the observed coalescence was dominated by inertial effects.

## 3. Coalescence of Drops on a Deformable Surface

Drops can behave differently on a deformable or soft substrate. For instance, when a drop is placed on a soft, deformable substrate, the surface tension along the contact line deforms the substrate [32]. Similarly, when drops merge on a deformable substrate, which is usually a liquid, the deformation and energy dissipation of the substrate influence the coalescence. Thus, drop coalescence on a deformable surface can be regarded as a special case of the three-phase drop coalescence on one confining surface. This section summarizes the coalescence of two types of drops on a liquid substrate: flat disks and liquid lenses.

### 3.1. Flat Disk Cases

**Figure 12:**
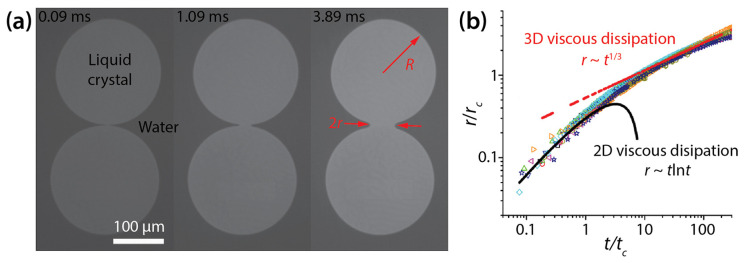
(**a**) Coalescence of thin liquid crystal drops on water (*μ_d_* ≈ 10^−8^ Pa·s·m, and *γ* = 69–158 pN) (top view). The seen surface of the drops was exposed to air, and the other surface was in contact with water. (**b**) Log–log scale plot of *r*(*t*). Here, $t_{c}=\sqrt{\mu_{d}^{3}R/\gamma^{2}\mu_{o}}$ and $r_{c}=\sqrt{\mu_{d}R/\mu_{o}}$. Adapted with permission from [33]. Copyright 2010 by the American Physical Society.

Delabre and Cazabat investigated the two-dimensional (2D) coalescence of thin liquid crystal drops (6CB member of the cyanobiphenyl series) that were formed on water and exposed to air (Figure 12a) [33]. This coalescence was driven by the line tension of the drops (*γ* = 69–158 pN). Since the thickness of the drops (*H* < 0.5 μm) was significantly smaller than the radius of the drops (*R* > 60 μm), the drops were essentially 2D. Additionally, inertia effects were negligible.

When scaled with *r*_c_ ($=\sqrt{\mu_{d}R/\mu_{o}}$) and *t*_c_ ($=\sqrt{\mu_{d}^{3}R/\gamma^{2}\mu_{o}}$), all data of *r*(*t*) collapsed onto one another regardless of *R*, *γ*, and *H* (Figure 12b). Here, *μ_d_* and *μ_o_* are the surface viscosity of the drop (*μ_d_* ≈ 10^−8^ Pa·s·m) and the bulk viscosity of the water substrate (*μ_o_* ≈ 10^−3^ Pa·s), respectively. Delabre and Cazabat observed that the bridge growth dynamics transitioned from an early 2D surface viscous dissipation to a later three-dimensional (3D) substrate viscous dissipation (Figure 12b).

In the former 2D viscous dissipation regime (*r* < *r*_c_), drop coalescence were governed by the balance between the driving force, which was the film pressure gradient due to line tension and the viscous dissipation of the drop. The scaling law for this regime was found to be *r*~−(*γ*/*μ_d_*)*t*ln(*γt*/*μ_d_R*) with the logarithmic correction of Hopper [34] (Figure 12b). In this regime, the coalescing drops did not feel the viscous resistance of the underlying water, and thus they were similar to coalescing infinite liquid cylinders.

In the latter 3D viscous dissipation regime (*r*_c_ < *r* << *R*), the growth dynamics of the bridge was governed by the balance between the driving force and the viscous dissipation caused by the water substrate. For this regime, Delabre and Cazabat found *r*/*R*~(*γ*/*μ_o_R*^2^)^1/3^*t*^1/3^ (Figure 12b). The scaling exponent of the second regime was smaller than that of the first regime, which shows that the bridge growth became slower, seemingly due to higher viscous dissipation of the water substrate.

### 3.2. Liquid Lens Cases

**Figure 13:**
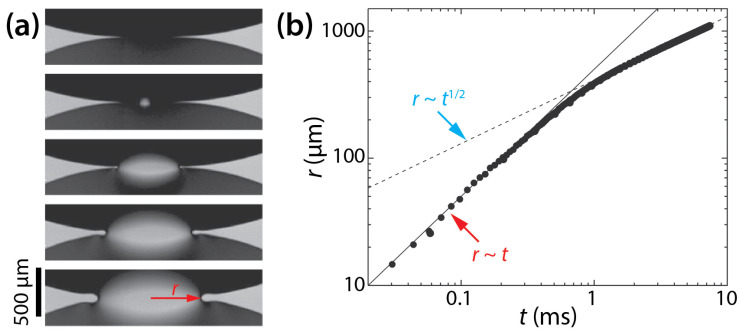
(**a**) Coalescence of dodecane lenses (*μ_d_* ≈ 1 mPa·s, and σ = 53 mN/m) on water (*θ* = 46°) (top view). Time interval: 0.748 ms. (**b**) Log–log scale plot of *r*(*t*). Adapted with permission from [36]. Copyright 2007 by the American Physical Society.

In contrast to flat disk drops of which the early-time coalescence is similar to the 2D coalescence of infinitely long liquid cylinders, the coalescence of liquid lenses is inherently 3D because of their varying height profile. Burton and Taborek investigated the dimensionality of drop coalescence between two circular dodecane lenses (*μ_d_* ≈ 1 mPa·s, and *σ* = 53 mN/m [35]) floating on water (*θ* = 46°) (Figure 13a) [36]. Burton and Taborek found two regimes of different scaling laws in the growth dynamics of the bridge (Figure 13b). In the first viscous regime, the scaling law was found to be *r*~*St*/*μ_d_*, where *S* is a spreading coefficient defined as *S* = *σ_wa_* − *σ_wo_* − *σ_oa_*. Here, subscripts *w*, *a*, and *o* denote water, air, and oil, respectively. In the second inviscid regime, the found scaling law was *r*~(*SR*/*ρ_d_*)^1/4^*t*^1/2^. Since the energetics of thin liquid lenses on water is described by *S*, *S* appeared in both scaling laws. The transition between the two regimes was characterized by a crossover length $l_{c}≅2\pi \mu_{d}\sqrt{R/\sigma \rho_{d}}$, which was 253 μm in their study. Burton and Taborek concluded that the growth of the bridge width of liquid lenses was similar to that of axisymmetric drops.

**Figure 14:**
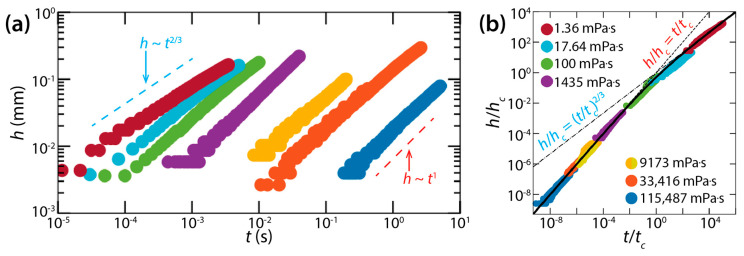
(**a**) Log–log scale plot of *h*(*t*) of the liquid bridge between drops of mineral oil (18 mPa·s < *μ_d_* < 115 Pa·s, *ρ_d_* = 850 kg/m^3^, and *σ* = 34 mN/m) or dodecane (*μ_d_* = 1.36 mPa·s, *ρ_d_* = 750 kg/m^3^, and *σ* = 25 mN/m) floating on water. (**b**) Superposition of data sets normalized with $t_{c}=288K_{i}\mu_{d}^{3}/K_{v}^{3}\rho_{d}\sigma^{2}\theta^{2}$ and $h_{c}=72K_{i}\mu_{d}^{2}/K_{v}^{2}\rho_{d}\sigma$. Adapted with permission from [37]. Copyright 2020 by the American Physical Society.

However, Hack et al. questioned the conclusion of Burton and Taborek that the bridge growth dynamics between liquid lenses was similar to that of unconfined drops [36], because the dynamics of drop coalescence is significantly affected by the geometry of drops and because liquid lenses are more like spherical caps than spheres [37]. As such, they tested two different liquids for liquid lenses: mineral oil (18 mPa·s < *μ_d_* < 115 Pa·s, *ρ_d_* = 850 kg/m^3^, *σ* = 34 mN/m) and dodecane (*μ_d_* = 1.36 mPa·s, *ρ_d_* = 750 kg/m^3^, *σ* = 25 mN/m). The contact angle and radius of those liquid lenses were *θ* = 26°–37° and *R* = ~2.5 mm, respectively. Assuming that the lenses were top-down symmetric with respect to their equator, Hack et al. measured the half-height of the bridge (*h*) (Figure 14a).

For the coalescence dynamics of liquid lenses, Hack et al. found the following. First, the coalescence of liquid lenses was governed by a self-similar growth of the bridge (in terms of the profile seen in the side view). Second, the early time of the coalescence consisted of two distinct regimes: a viscous regime and an inertial regime. Third, for each regime, the height growth of the bridge was well-represented by a scaling law without any adjustable parameters.

In the viscous regime, *h* grew linearly with *t* (i.e., *h*~*t*) (Figure 14a), and the rate of the bridge growth (i.e., d*h*/d*t*) decreased when *h* became of the order of the lens height (*H* ≈ 0.5 mm). Hack et al. found the dimensional bridge velocity to be $dh/dt=K_{v}\sigma \theta^{2}/4\mu_{d}$ with *K_v_* = 2.21, which agreed well with their experimental data. In the inertial regime, the found scaling law was *h*~*t*^2/3^ with $dh/dt={\left(9K_{i}\sigma \theta^{{}_{4}}/2\rho_{d}\right)}^{1/3}$ where *K_i_* was found to be 0.106.

Hack et al. also obtained crossover height *h_c_* and crossover time *t_c_* for the transition from the viscous regime to the inertial regime: $h_{c}=72K_{i}\mu_{d}^{2}/K_{v}^{2}\rho_{d}\sigma$ and $t_{c}=288K_{i}\mu_{d}^{3}/K_{v}^{3}\rho_{d}\sigma^{2}\theta^{2}$. It is noticeable that *h_c_* does not depend on *θ*, whereas *t_c_* does. When normalized with *h_c_* and *t_c_*, all data of *h*(*t*) superposed on a single curve despite several order of magnitude difference in *μ_d_* (Figure 14b). This master curve can be represented by the following empirical formula: *h*/*h_c_* = 1/[(*t*/*t_c_*)^−1^ + (*t*/*t_c_*)^−2/3^].

## 4. Coalescence of Drops in Hele-Shaw Cells

For studies of drops coalescence occurring between two parallel surfaces, a Hele-Shaw cell was used. The Hele-Shaw cell consists of two parallel surfaces with a cell depth (*D*) that is much smaller than the drop radius (i.e., *D* << *R*). Behaviors of drops in such a confining geometry are relevant to various applications in which a small amount of liquid is manipulated, such as microfluidics [23]. In addition, the flow velocity in the depth-wise direction is negligibly small compared to the velocity components in the other directions, which enables assuming quasi 2D flow [38]. Another advantage of using Hele-Shaw cells is that the geometry of drops is symmetric with respect to the mid-plane of drops. It needs to be pointed out that in the studies summarized in this section, drops did not make a direct contact with the wall of Hele-Shaw cells. Instead, a thin layer of the outer liquid existed between the drop and the wall [8]. Thus, these studies belong to the two-phase coalescence with two confining surfaces category and involved neither contact line dynamics nor surface wettability.

**Figure 15:**
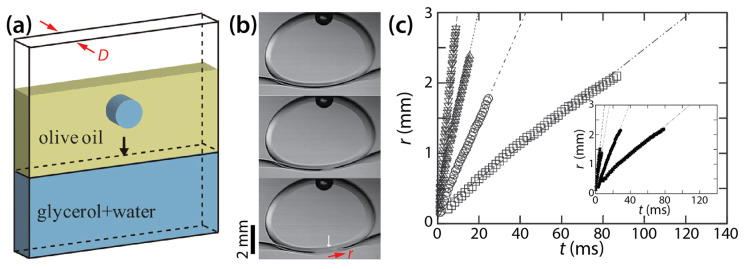
(**a**) The schematics of drop coalescence in a Hele-Shaw cell with a cell depth of *D*. (**b**) Coalescence between a drop of glycerol solution and the bath of the same glycerol solution (*D* = 2 mm) in olive oil (*μ_o_* ≈ 60 mPa·s). Time interval: 1.875 ms. (**c**) Linear scale plot of *r*(*t*) with *D* = 1 mm. Inset: *D* = 2 mm. *μ_d_* was ▽: 13.9 mPa·s, △: 53.1 mPa·s, ○: 125 mPa·s, and ☐: 280 mPa·s. Inset: ▼: 12.7 mPa·s, ▲: 40.2 mPa·s, ●: 128 mPa·s, and ■: 316 mPa·s. Adapted with permission from [39]. Copyright 2010 by the American Physical Society.

Okumura and his colleagues have long studied two-phase drop coalescence in Hele-Shaw cells. Eri and Okumura investigated bursting of thin liquid film formed between two immiscible liquids using a Hele-Shaw cell [39]. As shown in Figure 15a, they formed a bath of glycerol solution of various viscosity (*μ_d_* = 12.7–316 mPa·s) and another layer of olive oil of a fixed viscosity (*μ_o_* ≈ 60 mPa·s) and then placed a drop of the same glycerol solution as the bath in the oil. The achieved viscosity ratio (*φ* = *μ_d_*/*μ_o_*) ranged from 0.21 to 5.27.

When the drop reached the bath, a thin oil layer existed between them (the top image of Figure 15b). This thin film burst to initiate coalescence. Eri and Okumura measured the displacement of the moving tip of the oil film, which corresponded to the half width of the bridge (*r*). Figure 15c shows that *r* increased linearly with *t* (i.e., *r*~*t*) for two different cell depths regardless of *φ*, and that the slope decreased as the glycerol became more viscous (i.e., *φ* increased). Eri and Okumura derived the following relation of the bursting velocity (i.e., *V* = d*r*/d*t*; the slopes of lines in Figure 15c) by assuming that the viscous dissipation in the glycerol is balanced by the capillary driving force: *V*~*σ*/*μ_d_*.

**Figure 16:**
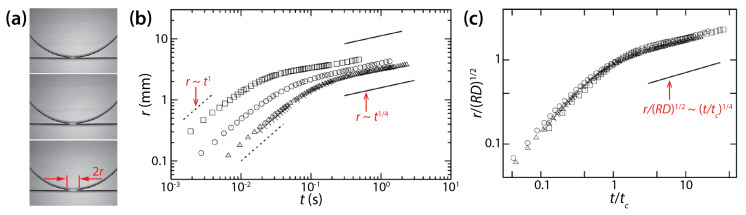
(**a**) Coalescence between a glycerol drop and a bath of the same liquid in low-viscosity PDMS oil (*μ_o_* ≈ 1 mPa·s). (**b**) Log–log lot of *r*(*t*). The values of *μ_d_*, *D*, and *R* are as follows: ☐: 62.9 mPa·s, 0.7 mm, 5.62 mm; ○: 289 mPa·s, 0.7 mm, 5.56 mm; △: 888 mPa·s, 1.0 mm, 4.13 mm; ×: 964 mPa·s, 1.0 mm, 4.32 mm. (**c**) Normalized plot of (**b**). Here, *t_c_* = *μ_d_R*/*σ*. Adapted with permission from [23]. Copyright 2011 by National Academy of Sciences.

Following the above study, Yokota and Okumura further investigated the dimensional crossover (from 3D to quasi 2D) in the coalescence dynamics between a quasi 2D glycerol drop (*μ_d_* = 63–964 mPa∙s, *ρ_d_* = 1.21–1.26 g/cm^3^, and *σ* = 20 mN/m) and a bath of the same glycerol in Hele-Shaw cells (*D* = 0.7 and 1.0 mm) [23,40]. In these studies, they used polydimethylsiloxane (PDMS; *μ_o_* ≈ 1 mPa·s and *ρ_o_* = 0.818 g/cm^3^), which was much less viscous than olive oil used in their previous study [39]. Since *μ_o_* << *μ_d_* (*φ* >> 1), the viscous effect of PDMS on coalescence was negligible, and the interface between the bulk glycerol and PDMS remained horizontal when the glycerol drop reached the glycerol bath (Figure 16a).

Yokota and Okumura identified two regimes of drop coalescence dynamics (Figure 16b). In the early time regime, the diameter of the bridge was smaller than the cell depth of the Hele-Shaw cell (i.e., *r* ≲ *D*/2 and *t* ≲ *μ_d_D*/*σ*). Thus, there was enough space for the liquid bridge to grow and this growth was not limited by the walls of the Hele-Shaw cell, so the bridge growth was 3D. They found the following scaling law for the early time regime: *r*/*D* ≃ *t*/(*μ_d_D*/*σ*) (i.e., *t_c_* = *μ_d_D*/*σ*) by balancing a gain in surface energy with a viscous dissipation localized in the bridge. Data shown in Figure 16b in this regime collapsed well on a straight line when normalized based on the found scaling law. This initial linear growth of the bridge agrees with their previous study (i.e., *V* = *σ*/*μ_d_*) [39] and the well-established 3D viscous coalescence of unconfined drops.

As the liquid bridge grew further and touched the cell wall, the second regime appeared with the following scaling law: $r/\sqrt{RD}={\left[t/\left(\mu_{d}R/\sigma \right)\right]}^{1/4}$ (i.e., *t_c_* = *μ_d_R*/*σ*) (Figure 16b). The data in this regime superposed well on a straight line when normalized based on the found scaling law (Figure 16c). This scaling law for the later time regime ($\sqrt{RD}$ ≲ *r* ≲ *R* and *t* ≳ *Rμ_d_*/*σ*) was also found from the balance between the gain in surface energy and the viscous dissipation. In this regime, the bridge did not grow in the depth-wise direction of the Hele-Shaw cell. Also, this viscous dissipation occurred in the thin oil film between the liquid bridge and the cell wall. Thus, the found scaling law for the later time regime is for quasi 2D disk drops and is different from the one for unconfined drops. It is noticeable in Figure 16c that the scaling law for the later time regime resulted in a good superposition of data sets, even for the early time regime.

Dolganov et al. observed the same power–law scaling relation (*r*~*t*^1/4^) as Yokota and Okumura for the coalescence of two drops of liquid crystal E7 occurring in a Hele-Shaw cell (*D* = 5–50 μm) [41]. In their study, coalescence occurred between two quasi-2D drops, not between a drop and a bath. Following the above scaling law of Yokota and Okumura, Dolganov et al. scaled their data using *r*/*R*~(*t*/*t_c_*)^1/4^ and determined the *t_c_* value of each data set so that the data followed the scaling law. Their data superposed well on each other for the middle stage of coalescence (0.5 < *r*/*R* < 1), which appears to correspond to the later time regime of Yokota and Okumura [23]. Dolganov et al. observed that determined *t_c_* values increased with *R* following *t_c_*~*R*^3^, which differs from Yokota and Okumura (*t_c_*~*R*) [23].

Recently, Koga and Okumura investigated drop coalescence between a glycerol drop (*μ_d_* = 23.6–388 mPa·s, and *ρ_d_* = 1.18–1.25 g/cm^3^) and a bath of the same glycerol in PDMS (*μ_o_* = 4875 and 9750 mPa·s, and *ρ_o_* = 0.975 g/cm^3^) in the Hele-Shaw cell (*D* = 2 mm) [42]. The achieved *φ* values ranged from 0.0024 to 0.08, which means that in all experiments, the drops were less viscous than the outer liquid (i.e., *φ* < 1). The interfacial tension between the two liquids was practically constant: *σ* = 30.2 ± 2.3 mN/m.

Koga and Okumura found that a cylindrical bridge of radius *r* and length *l* was formed between the drop and the bath. The bridge remained cylindrical for *r* << *D*/2, which corresponded to the early-time regime of Yokota and Okumura [23]. Similar to their previous studies [23,39,40], Koga and Okumura found *r*~*t* for *t* ≲ 0.05 s. For this regime, they found the following relation: *r*/*l* = *kt*/*t_c_*, where $t_{c}=\sqrt{\rho_{d}l^{3}/\sigma }$ and *k* is a dimensionless coefficient. When the data in this regime were normalized with the found scaling law, they superposed well on each other with *k* = 0.013 ± 0.002. Based on these results, Koga and Okumura concluded that the early time regime of the drop coalescence in the Hele-Shaw cell with *φ* < 1 was governed by the inertia of the drop, and the dynamics did not rely on the viscosities. Their conclusion was in contrast to the study of Paulsen et al. on the coalescence of unconfined drops [17] stating that the early time dynamics of bridge growth is always governed by the viscosity of drops regardless of *φ*.

**Figure 17:**
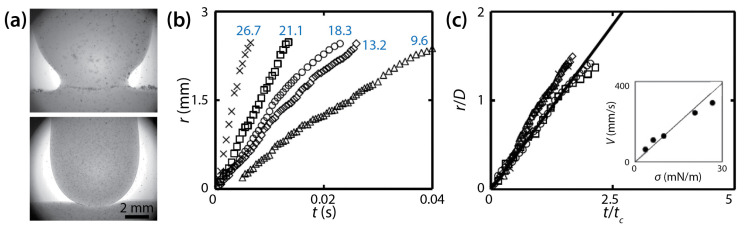
(**a**) Drop coalescence between a glycerol drop and a bath of the same liquid (76% *v*/*v*, *μ_d_* = 54 mPa∙s, and *ρ_d_* = 1210 kg/m^3^) in a low-viscosity oil (*μ_o_* = 1.75 mPa∙s, and *ρ_o_* = 8.4 kg/m^3^) in a Hele-Shaw cell (*D* = 1.25 mm). Top: without Span 80. Bottom: with Span 80 (0.015% *w*/*w*). (**b**) Linear scale plot of *r*(*t*) with different *σ* values (blue numbers: mN/m). (**c**) Normalized plot of (**b**). Here, *t_c_* = *μ_d_D*/*σ*. Inset: Averaged speed of the neck growth. The solid black line in the inset represents the fitted slope for α = 0.7. Adapted from [38].

Chinaud et al. studied the effect of a surfactant on drop coalescence in a Hele-Shaw cell of *D* = 1.25 mm [38]. Aqueous glycerol solution (76% *v*/*v*, *μ_d_* = 54 mPa∙s, and *ρ_d_* = 1210 kg/m^3^) was used for the drop and bath, and a low-viscosity oil (Exxsol D80, *μ_o_* = 1.75 mPa∙s, and *ρ_o_* = 804 kg/m^3^) was used for the outer liquid (*φ* ≈ 31). Span 80 was added to the oil as a surfactant to change *σ* (9.6–26.7 mN/m). The addition of the surfactant was found to modify the shape of the approaching interface of the drop (Figure 17a). Without the surfactant, the bridge was located above the initial flat interface level of the bath during the entire coalescence process. However, with the surfactant, the bridge was located below the initial interface level.

Chinaud et al. observed that the bridge grew linearly with time (i.e., *r*~*t*) irrespective of *σ* (Figure 17b), and that this linear trend continued until the bridge grew beyond the depth of the cell (i.e., during the 3D growth for *r*/*D* < 1). This linear trend agrees with the studies of Okumura and his colleagues [23,39]. It was noticed that the slope of *r* (i.e., *V*) decreased at *r*/*D* ≈ 1, which indicated that the growth of the bridge became slower as it transitioned from 3D to quasi 2D. Although this change in the slope agrees with Yokota and Okumura [23], Chinaud et al. did not show whether the later time regime followed *r*~*t*^1/4^.

Figure 17b shows that the slopes of the data points (i.e., *V*) decreased with the surfactant concentration. This result shows that the growth of the liquid bridge became slower with high concentrations of the surfactant because the driving force for coalescence decreased with lower *σ*. Using the following scaling law of Eri and Okumura [39]: *V* = *ασ*/*μ_d_*, where *α* is a correction factor, Chinaud et al. determined the value of *α* to be 0.7 (the inset of Figure 17c) assuming that the local surfactant concentration in the bridge region was two times of that of the bulk during the early stage of coalescence. With the found *α* value, all curves superposed on a single line (Figure 17c), which suggests that the assumption was valid. Therefore, Chinaud et al. concluded that the local concentration of the surfactant at the bridge region should be considered properly for understanding the bridge growth dynamics of coalescing drops.

## 5. Computational Studies

Although in the current review we mainly focus on experimental studies on the coalescence of confined drops, we briefly summarize computational studies on the topic because experimental studies can be complemented by computational simulations that enable the easy modulation of fluid properties pertaining to the phenomenon [43,44,45,46,47].

Sui et al. simulated coalescence between two identical drops resting on a partially wetting surface, employing a diffuse-interface method and a volume-of-fluid method [43]. For inertial coalescence (Oh = $\mu_{d}/\sqrt{\rho_{d}\sigma R}$~10^−3^) with a wide range of *θ* (*θ* < 90°), Sui et al. found *h*/*R*sin*θ*~(*t*/*t_c_*)^2/3^ and *r*/*R*sin*θ*~(*t*/*t_c_*)^1/2^. Here, *R* is the radius of curvature of the initial drop, and *t_c_* = (*ρ_d_R*^3^sin^3^*θ*/*σ*)^1/2^. The scaling exponent of *r* (*n* = 1/2) agrees with the experimental result of Kapur and Gaskell [27]. Also, Sui et al. found that the *n* value of *h*(*t*) changed from 2/3 to 1/2 at a certain critical time and that the critical time decreased to 0 as *θ* approached 90°. This means that *h*~*t*^1/2^ for *θ* = 90°. This change in *n* for *h* agrees with Eddi et al. [28]. For Oh = 0.1, where viscous effects are no longer negligible, Sui et al. found *h*~*t*^0.88^ for *θ* = 70°. This result agrees with the result of Lee et al. [18] of which the Oh range was 0.5–0.9.

Ahmadlouydarab et al. simulated coalescence of sessile drops on a solid surface with a linear gradient of *θ* using a many-body dissipative particle dynamics method [44]. For two identical drops that experienced *θ* changing from 82° to 90°, they found *r*~*t*^0.55^ and *h*~*t*^0.54^ (Oh ≈ 0.5). Also, Ahmadlouydarab et al. found that *r* grew faster than *h* on a wettable surface (*θ* < 90°) and *h* grew faster than *r* on a non-wettable surface (*θ* > 90°). In their simulation on coalescence between drops of different sizes, the smaller drop was merged into the bigger drop. That was because the greater capillary driving force of the smaller drop due to the larger curvature caused faster flow inside the smaller drop. For the coalescence between unequal-sized drops, they found that the *n* values of *r*(*t*) and *h*(t) were around 0.5 while varying depending on the drop sizes.

Pawar et al. employed the lattice Boltzmann method to simulate inertial coalescence between two sessile drops for three cases (Oh ≈ 0.04): equal-sized drops on a surface with homogeneous wettability (*θ* = 48°, 62°, 77°, and 90°), unequal-sized drops on a surface with homogeneous wettability (*θ* = 48°, 62°, and 90°), and drops having different *θ* (*θ* = 90° for one drop, and *θ* < 90° for the other drop) [45]. For the first case, they found that *h* grew faster with *θ*. For this result, Pawar et al. provided the following explanation. For a fixed volume of drops, as *θ* increases, the drop radius decreases, which increases the internal pressure of the drop, and the radius of curvature of the bridge simultaneously decreases, which results in higher capillary pressure difference. As a result, the drop liquid flows faster toward the bridge. Similar to Eddi et al. [28], Pawar et al. also found that for *h*(*t*), *n* = 2/3 for *θ* < 90° and *n* = 1/2 for *θ* = 90°. For the second case, they observed that the smaller drop was absorbed into the bigger drop, which agrees with the simulation result of Ahmadlouydarab et al. [44], because of faster liquid flow from the smaller drop toward the bridge compared to the bigger drop. It is noticeable that the scaling law for equal-sized drops is valid for unequal-sized drops. For the last case, Pawar et al. found that the growth dynamics of the bridge depended on the lower *θ* because they obtained *n* = 2/3 for all cases. This result of Pawar et al. does not agree with the finding of Hernández-Sánchez et al. [25], because the latter found *n* = 1 for asymmetric drops of *θ_L_* = 46° and *θ_R_* = 13°. The significant difference in *θ* needs to be considered when comparing the two studies.

## 6. Summary, Challenges, and Outlook

Table 1, Table 2 and Table 3 summarize the scaling laws for the bridge growth of confined drop coalescence for three different categories: three-phase coalescence on a solid surface, three-phase coalescence on a deformable surface, and two-phase coalescence in a Hele-Shaw cell (Figure 2). The tables clearly demonstrate that the scaling laws differ depending on the type of the confinement and that the dynamics of coalescence is influenced by the shape or geometry of the drops. In particular, on a solid surface, the shape of sessile drops is governed by the surface wettability of the underlying rigid surface. As such, the coalescence dynamics of drops change as the contact angle of the drop (*θ*) changes.

In the case of wetting drops (*θ* < 90°), they are in the shape of spherical caps with a small polar angle, and their liquid bridge between the drops is always in contact with the surface, even from its birth. As such, the three-phase coalescence of wetting drops on a solid surface differs from the two-phase coalescence of unconfined drops as follows. First, the growth dynamics of the bridge appear to be influenced by the no-slip boundary condition of the surface, which imposes more viscous resistance throughout the coalescence process. Second, contact lines exist between the bridge and the solid surface, and the bridge growth seems to involve the pinning and depinning of the contact line. Lastly, the radii of curvature of the drops and bridge change depending on *θ*, which in turn changes the capillary pressure difference between the drop and the bridge [45].

As a result of interactions between the bridge and the surface, the liquid bridge grows differently in the horizontal direction (i.e., parallel to the solid surface) and the normal direction (i.e., perpendicular to the surface). Table 1 shows that the *n* values for *r*(*t*) were found to be around 1/2 regardless of *θ*, whereas various *n* values were found for *h*(*t*) depending on *θ*. Also, *n* values are different between *r*(*t*) and *h*(*t*) for *θ* ≤ 56° [18,24,25,26] but similar for 56° < *θ* < 90° [27,28]. These differences in *n* illustrate different growth dynamics between *r* and *h*.

Here, it needs to be noted that discrepancies exist between studies for similar θ values. For instance, Hernández-Sánchez et al. found *h*~*t*^1^ for *θ* = 22° [25], whereas Lee et al. found *h*~*t*^0.64^ for *θ* = 22° [18]. For *θ* = 56°, Lee et al. found *r*~*t*^1/4^ and *h*~*t*^0.86^ [18] while Kapur and Gaskell found *r*~*t*^0.5^ and *h*~*t*^0.5^ for a dynamic contact angle range including 56° [27]. These disagreements may indicate a possibility that the examined regime of coalescence could be different. In the case of Lee et al. and Kapur and Gaskell, their Oh ($=\mu_{d}/\sqrt{\rho_{d}\sigma R}$) values are quite different: 0.4–0.5 for the former and 10^−3^–10^−2^ for the latter. Clearly, the coalescence that Kapur and Gaskell examined was inertia-dominated because Oh << 1. In contrast, the coalescence that Lee et al. examined was not free of viscous effects because Oh > 0.1. For a similar Oh (=0.1), Sui et al. found *h*~*t*^0.88^ [43], which is very close to the result of Lee et al. [18]. These comparisons suggest that Oh and any relevant dimensionless numbers should be considered when investigating the coalescence of sessile drops on a wettable solid surface.

Another possible reason for the discrepancy can be different ways of determining the initial moment of drop coalescence (i.e., *t* = 0 for *r* = 0 and *h* = 0). Only a few studies among the reviewed literature clearly indicated how the initial moment was determined. For instance, Lee et al. assumed that the moment of the deposition of the second drop to be the initial moment and admitted that it was very hard to pinpoint the initial moment [18]. Hack et al. defined the initial moment as the first frame where the liquid bridge became visible [37], which means that the true initial moment was before the identified first frame due to their imaging resolution. Eri and Okumura defined the initial moment as the instant when the oil film began to burst and admitted that the initial moment could be shifted at most 1/2000 s [39]. We found that *n* values changed depending on how the initial moment was determined, especially for the early time of drop coalescence (personal communication with Okumura). The initial moment is usually determined indirectly using captured high-speed images of the liquid bridge, which is limited by the temporal and image resolution of the videography setting. It would be more reliable to determine the initial moment using more direct ways, such as the electrical method of Case [48,49].

In the case of non-wetting drops (*θ* > 90°), drops merge similar to unconfined drops because they are in the shape of spheres with a spherical dome removed (i.e., spherical caps with a large polar angle). Their liquid bridge is formed away from the solid surface, and thus its growth is not affected by the no-slip boundary condition of the surface and the dynamics of the contact line. As a result, the scaling exponent of *r*~*t^n^* is around 1/2 [9,19,30], which agrees with that of the inertial regime for unconfined drop coalescence [9,10,11,12,13,14,15,16]. For the unconfined drop coalescence, the viscous regime of *r*~*t*^1^ precedes the inertial regime. Thus, the similarity between the two cases suggests that the viscous regime may also exist in the very early time of the coalescence of non-wetting drops. Also, for a very late time of the coalescence, the liquid bridge between the non-wetting drops will hit the surface, and its growth may be significantly slowed by the no-slip condition of the solid surface depending on the initial distance from the bridge to the surface. If this happens, the scaling exponent would decrease further.

For drops floating on water in air, which is categorized as three-phase coalescence on a deformable surface (Table 2), their coalescence causes the underlying liquid to flow. As a result, the growth of their liquid bridge is hampered by the viscous energy dissipation of the liquid. Although impeded by the water, the drop coalescence on the liquid substrate is faster than that on a solid substrate because the no-slip boundary condition of the solid surface induces greater resistance. The coalescence of liquid lenses is observed to be about five times faster than drop coalescence on a solid surface [37]. The viscous energy dissipation of the liquid substrate seems to become more dominant for the later time of coalescence because the early time coalescence dynamics of flat disk-shaped drops is similar to that of 2D liquid cylinders [33]. In contrast, the studies on the coalescence of liquid lenses did not consider the viscosity effect of the underlying water [36,37]. Since all the reviewed studies for this category used water as the deformable substrate [33,36,37], testing liquid substrates of different viscosity and other types of soft substrates [50] is required for advancing understanding of the effect of deformable substrates on the coalescence of sessile drops.

Burton and Taborek [36] and Hack et al. [37] both used dodecane of similar viscosity (*μ_d_* ≈ 1 mPa·s) for liquid lenses and examined the coalescence for a similar time range (*t* ≈ 0.01–10 ms). The former found that the scaling exponent of *r*(*t*) changed from 1 to 1/2 during the course of coalescence (Figure 13b), and the latter found that the scaling exponent of *h*(*t*) transitioned from 1 to 2/3 (Figure 14b). Therefore, the bridge between dodecane liquid lenses grew differently in the horizontal and normal direction, similar to sessile drops coalescing on a rigid surface.

For two-phase drop coalescence of drops in the Hele-Shaw cell, the growth of the liquid bridge is limited by the cell wall because the gap depth of the Hele-Shaw cell is much smaller compared to the size of drops. During the early time during which the diameter of the liquid bridge is smaller than the cell depth (*d* < *D*), the liquid bridge grows three-dimensionally, not limited by the cell wall. Eventually, the liquid bridge reaches the cell wall, and it cannot grow anymore in the depth-wise direction of the Hele-Shaw cell. As a result, the liquid bridge grows only in the width-wise direction, which is quasi 2D. This shift in dimensionality is represented by the change in the scaling exponent of the scaling law (Figure 16b).

Using the Hele-Shaw cell provides the following advantages in comparison to using one confining surface. First, drops formed in the Hele-Shaw cell are symmetric with respect to the equator of the drops or the mid-plane of the cell, which is advantageous for theoretical modeling and imaging of the growing liquid bridge. Second, the outer fluid can be easily changed to a more viscous liquid. In all studies reviewed for sessile drops on a plane, the outer fluid was air, and thus the viscosity effect of the outer fluid was negligible. In contrast, Okumura and his colleagues could achieve a wide range of viscosity ratio (*φ* = *μ_d_*/*μ_o_*) by filling their Hele-Shaw cell with an outer fluid and then creating a drop [23,39,42]. Thus, the Hele-Shaw cell enables investigating how the growing liquid bridge feels the no-slip boundary condition of the cell wall via the viscosity effect of the outer fluid [6].

As reviewed so far, the coalescence of confined drops poses more complicated interfacial fluid dynamics challenges but has been less studied compared to that of unconfined drops. We have categorized the phenomenon, as shown in Figure 2, and reviewed the current literature on three-phase coalescence with one confining surface, which was wettable and not-wettable solid surfaces and water, and two-phase coalescence between two parallel confining surfaces with a small gap (i.e., Hele-Shaw cell). To promote a more holistic understanding of the effect of the confining surfaces on drop coalescence, future studies will need to investigate drop coalescence on soft deformable solid substrates and liquid substrates of different viscosity, and three-phase coalescence in Hele-Shaw cells, and analyze and compare results in terms of relevant dimensionless numbers.

## Figures and Tables

**Table 1:** Power–law scaling relations of drops coalescing on a single solid surface (three phase coalescence).

Authors	Contact Angle (*θ*)	Characteristic Scale		Scaling Law	
		Width	Height	Width (*d* or *r*)	Height (*h*)
Ristenpart et al. [24]	≈0°	*t_c_* = *μ_d_R*^4^/*σH*^3^		*d*/*R* ~ (*t*/*t_c_*)^0.53^	
Hernández-Sánchez et al. [25]	22°				*h*~*t* d*h*/d*t*~*θ*^4^
	*θ_L_* = 46° *θ_R_* = 13°				*h*~*t*
Narhe et al. [26]	*θ_a_* = 35° *θ_r_* = 25°	*t_c_* = *μ_d_R*/*σ*		*d*/2*R*~*θ*(*t*/*t_c_*)^1/2^	*h*/*h*_∞_~*t*/*t_c_*
Lee et al. [18]	10°, 24°, 27°, and 56°	*t_c_* = 3*μ_d_R*/4*σ*tan^4^*θ*		*r*~(*σh_o_*^3^/*μ_d_*)^1/4^*t*^1/4^	*h*/*R*~(*t*/*t_c_*)*^n^* *n* = 0.51 for *θ* = 10° *n* = 0.64 for *θ* = 24° *n* = 0.67 for *θ* = 27° *n* = 0.86 for *θ* = 56°
Kapur and Gaskell [27]	*θ_a_* = 64°/*θ_r_* = 58° *θ_a_* = 56°/*θ_r_* = 49°	*t_c_* = *μ_d_R*/*σ*	$t_{c}=\sqrt{\rho_{d}R^{3}/\sigma }$	*r*/2*R*~(*t*/*t_c_*)*^n^* *n* = 0.42–0.57	*h*/*R*~(*t*/*t_c_*)^1/2^
Eddi et al. [28]	73°			*d*~*t*^2/3^	*h*~[*σ*/*ρ_d_*(π/2-*θ_a_*)]^1/3^*t*^2/3^
	81°, 84°		$t_{c}=\sqrt{\rho_{d}R^{3}/\sigma }$		*h*/*R*~(*t*/*t_c_*)^2/3^
	90°		$t_{c}=\sqrt{\rho_{d}R^{3}/\sigma }$		*h*/*R*~(*t*/*t_c_*)^1/2^
McCraney et al. [30]	115°–143°			*d*~*t*^0.4^	
Menchaca-Rocha et al. [9]	≈160°			Early time (*t* < 0.3 ms): *r*~*t*^0.41^ Later time (*t* > 1 ms): *r*~*t*^0.55^ Overall: *r*~*t*^1/2^	
Wang et al. [19]	162°–165°	$t_{c}=\sqrt{\rho_{d}R^{3}/\sigma }$		*r*/*R*~(*t*/*t_c_*)^1/2^	

**Table 2:** Power–law scaling relations of drops coalescing on a single deformable surface (three phase coalescence).

Authors	Contact Angle (*θ*)	Characteristic Scale	Scaling Law	
			Width (*d* or *r*)	Height (*h*)
Delabre and Cazabat [33] (flat discs)		$r_{c}=\sqrt{\mu_{d}R/\mu_{o}}$ $t_{c}=\sqrt{\mu_{d}^{3}R/\gamma^{2}\mu_{o}}$	Early time (*r* < *r*_c_)*: r*/*r_c_*~(*t*/*t_c_*)ln(*t*/*t_c_*) Later time (*r*_c_ < *r* << *R*)*: r*/*r_c_*~(*t*/*t_c_*)^1/3^	
Burton and Taborek [36] (liquid lenses)	46°		Early time: *r*~*St*/*μ_d_* Later time: *r*~(*SR*/*ρ_d_*)^1/4^*t*^1/2^	
Hack et al. [37] (liquid lenses)	26°–37°	$h_{c}=72K_{i}\mu_{d}^{2}/K_{v}^{2}\rho_{d}\sigma$ $t_{c}=288K_{i}\mu_{d}^{3}/K_{v}^{3}\rho_{d}\sigma^{2}\theta^{2}$		Early time: *h*/*h^c^* = *t*/*t^c^*, $dh/dt=K_{v}\sigma \theta^{2}/4\mu_{d}$ Later time: *h*/*h_c_* = (*t*/*t_c_*)^2/3^, $dh/dt={\left(9K_{i}\sigma \theta^{4}/2\rho_{d}\right)}^{1/3}$

**Table 3:** Power–law scaling relations of drops coalescing in a Hele-Shaw cell (two phase coalescence).

Authors	Viscosity Ratio (*φ*)	Characteristic Scale	Scaling Law
Eri and Okumura [39]	0.21–5.27		*r* ~ *t* d*r*/d*t* ~ *σ*/*μ_d_*
Yokota and Okumura [23]	63–964	Early time: *t_c_* = *μ_d_D*/*σ* Later time: *t_c_* = *μ_d_R*/*σ*	Early time (*t* *≲* *μd*/*σ*): *r*/*D* ≃ *t*/*t_c_*, d*r*/d*t* = *σ*/*μd* Later time (*t* ≳ *μdR*/*σ*): $r/\sqrt{RD}={\left(t/t_{c}\right)}^{1/4}$
Dolganov et al. [41]		*t_c_*~*R*^3^	*r*/*R* ~ (*t*/*t_c_*)^1/4^ (0.5 < *d*/2*R* < 1)
Koga and Okumura [42]	0.0024–0.08	$t_{c}=\sqrt{\rho_{d}l^{3}/\sigma }$	Early time (*r* << *D*/2): *r*/*l*~*t*/*t_c_*
Chinaud et al. [38]	31	*t_c_* = *μ_d_D*/*σ*	*r*/*D*~*t*/*t_c_*

## Data Availability

No new data were created or analyzed in this study. Data sharing is not applicable to this article.
